# Promoting healthy ageing through light volleyball intervention in Hong Kong: study protocol for a randomised controlled trial

**DOI:** 10.1186/s13102-019-0151-7

**Published:** 2020-01-28

**Authors:** Ka Man Leung, Pak-Kwong Chung, Aileen W. K. Chan, Lynda Ransdell, Parco Ming Fai Siu, Ping Sun, Jinjin Yang, Tie Cheng Chen

**Affiliations:** 1 Department of Health and Physical Education, Education University of Hong Kong, Education University of Hong Kong, 10 Lo Ping Rd, Ting Kok, Tai Po, Hong Kong; 2 Department of Sport and Physical Education, Hong Kong Baptist University, Kowloon Tong, Hong Kong; 3 The Nethersole School of Nursing, The Chinese University of Hong Kong, Hong Kong, Hong Kong; 4 College of Health and Human Services, Northern Arizona University, Flagstaff, USA; 5 Division of Kinesiology, School of Public Health, The University of Hong Kong, Pok Fu Lam, Hong Kong Hong Kong; 6 Sport Education and Training, Beijing Sport University, Beijing, China; 7 Exercise Science Department, Southern Connecticut State University, New Haven, USA; 8 School of Physical Education and Sport Science, College of Sports Science, Fujian Normal University, Fuzhou, China

**Keywords:** Physical activity, Age-related fitness degradation, Adapted sport, Ageing

## Abstract

**Background:**

Our pilot study has demonstrated improvements in health outcomes through participation in a new sport, light volleyball (LVB), among older adults. In response to the promising results of the LVB pilot study and the priority of allocating resources to the prevention of age-related fitness degradation by the Hong Kong (HKG) government, the present study aims to investigate the effectiveness of a LVB intervention on physical and psychological health attributes among older adults at a larger scale in HKG.

**Methods/design:**

This study will apply both quantitative and qualitative methods with a large sample (approximately 315 participants). We will adopt a randomized controlled trial (RCT) design to further evaluate the effectiveness of a LVB intervention on health outcomes against a comparison group, Tai Chi (TC), and a control group (C). Older adults will be eligible to join the intervention if they are (a) aged 65 years and above; (b) living in the community independently; (c) absent of diagnosed cognitive impairment; (d) not regular participants in a structured PA program for two years preceding the study; and (e) able to achieve a passing score on the Timed-up-and-go test (TUG) and Abbreviated Mental Test (AMT).

About 315 participants will be randomly assigned into 3 groups in 1:1:1 ratio. LVB group participants will receive 16-week LVB program; TC group will utilize a simplified 24-form Yang Style TC, and C group participants will be instructed to maintain their normal daily activity and join regular non-exercise social gatherings. Measurements will be collected before and after the intervention, and 6 months and 12 months after completion of the intervention.

**Discussion:**

This intervention, if effective, will enhance older adult’s physical and psychological health, and provide the data and evidence to support policymaking in relation to future PA promotion for older adults.

**Trial registration number:**

ChiCTR1900026657.

## Background

### Health needs of the local community

There is a continuous ageing trend in the population of HKG. In the forthcoming decades, the number of people in HKG aged 65 or above will increase to 2.16 million by 2031 and 2.56 million by 2041 [1]. Similar ageing trends are found in CHN [2]. According to the Prediction Report on the Development Trend of Population Aging in CHN, there were 241 million Chinese citizens aged 60 or above in 2017, about 17.3% of China’s total population. This figure will peak at 487 million, or nearly 35% in 2050. The ageing population presents substantive problems for social services such as reductions in the working-age population and increasing fiscal pressure on healthcare, social welfare, and other services for older adults [2, 3]. Owing to the effects of net medical inflation, population growth and ageing, and assuming the service enhancement continues with the historical trend, Hong Kong’s recurrent social welfare and health expenditure as a percentage of nominal GDP would increase from $56.9 billion and $52.4 billion in 2014 and 2015, respectively, and to $523.3 billion and $563.6 billion in 2041 and 2042, respectively [4]. A structural financial deficit may then strike within a decade in HKG due to the gradual increase in older adult’s social and health expenditure and reduction in working-age population [2, 3].

Despite the fact that the benefits of participating in physical activity (PA) are well documented [5], only about 30% of older adults aged 60 to 69 years, participated in sufficient PA in HKG and CHN [6, 7]. Age-related fitness degradation was also found in Chinese older adults [8]. Responding to the population ageing and its social implications, Governments [3, 9] have initiated programs to promote active ageing to enable older adults to stay active and remain healthy (e.g. offering older adults with diversified courses on an on-going basis, training for the trainers). Therefore, developing effective interventions (e.g., light volleyball in this study) is critical for promoting active lifestyles among older adults that can reduce the number of elderly adults becoming frail at an early stage.

Chase et al. [10] conducted a systematic review and meta-analysis to determine the effects of supervised PA (e.g., resistance exercise and aerobic PA) interventions on physical functioning among older adults and concluded that supervised PA interventions were effective at improving physical functioning (d = .45). Limitations of the reviewed twenty-eight studies were identified, as 1) few related studies from Asia; 2) small sample sizes with median control group size of 19 participants; 3) intervention duration did not meet the current World Health Organization (WHO) global recommendation of 150 min of PA per week [5]; and 4) lacking of control group or RCT study design.

In CHN, a systematic review [11] found promising evidence of the positive health benefits (e.g. mental and physical health, quality of life, and balance) of traditional Chinese sports [e.g., Tai Ji Quan (or Tai Chi), and Qigong] and PA among older Chinese adults. The limitations of previously reviewed studies suggest that larger scale interventions with prospective follow-ups, RCTs with representative and sufficiently powered samples, and the use of valid and reliable outcome measures are recommended for future research. Importantly, Guo and colleagues [11] mentioned that other than these mainstream activities, there are more than 900 types of Chinese sports/PAs (including the newly developed sport, LVB) in existence [12]. More research is needed to explore and evaluate the health impact of these non-mainstream activities. This is in line with the suggestion of Blewitt and Chockalingam [13], that further investigation should be conducted to identify new PA for older adults to gain the benefits of being physically active.

Qualitatively, Franco [14] reviewed the existing literature on older adult’s opinions on participation in PA. Among the 132 studies included, older adults valued PA participations and programs that emphasized “interaction with peers such as preferred group-based activity”, “professional instruction”, and “physical limitations such as discomfort and concerns about falling”. Similar themes about older adult’s perspectives on currently existing PA programs/interventions were found in Van Dyck et al. [15]. Furthermore, older adults who were interviewed suggested new PA (e.g., aquafitness or LVB in this study), not just regular activities like walking and cycling, should be selected as intervention activities. Likewise, the Hong Kong older adults also suggested interventions “providing more sport training classes (19.4%)” can help the promotion of Sport for All [7]. Based on the aforementioned considerations, we found that there is a gap between research and service relative to promoting active ageing.

### Scientific evidence supporting the strategies to address the needs proposed in this project

Among different types of PA, LVB is a new PA (also can be a sport) modified from traditional volleyball that may help reduce fitness-related degradation and increase PA among older adults. In contrast to traditional volleyball, LVB uses a lighter weight (LVB 150 g vs. traditional volleyball 250 g) and larger sized (LVB 80 – 83 cm in circumference vs. traditional volleyball 65-67 cm) ball. The LVB ball travels in the air at a lower velocity and for a longer time, which increases the playability and rally time among players during the games. It thus makes the LVB game more accessible to those with ageing-associated degradation (i.e. slow movements and reaction time, etc.). LVB, as a modified PA, is preferable to other traditional sports (even as it is adapted to older adults) because LVB is a non-contact and team-effort sport and is played with two teams separated by a net. These LVB features reduce the older adults’ likelihood of injury, such as falling, and these features address the recommendations of Franco [14] regarding factors that facilitate older adult’s participation in PA. Additionally, the LVB playing court (i.e., a standard-sized badminton court) is smaller than a standard volleyball court, which enables the promotion of LVB in sport facilities that normally have limited space in HKG. Moreover, with the popularity of the China National Volleyball team and annual HKG professional volleyball competition (i.e., FIVB Volleyball Nations League), volleyball is a traditional and popular sport in HKG. Volleyball is one of the sports taught in the school Physical Education curriculum in HKG and CHN. All of these factors favor LVB promotion in HKG in the future.

Studies in CHN indicated that older adults gained physical and psychological health benefits from regular LVB practice [16]. However, some limitations were found in these studies, such as unclear fitness component measurements and lack of control groups. In 2018, Leung et al. [17] conducted a quasi-experimental intervention to evaluate the effects of a 15-week LVB intervention on physical and psychological attributes among 78 Hong Kong older adults (≥ 60 years) by comparing LVB to a modified form of physical activity, rouliqiu (RLQ), and to a control group. Compared to the control group, LVB participants experienced significant improvements in agility, cardiovascular endurance, upper and lower extremity muscle strength, and physical activity enjoyment. Compared to their RLQ counterparts, participants in the LVB group demonstrated greater cardiovascular endurance, upper extremity muscle strength, and physical activity enjoyment. This pilot study suggested that the effectiveness of LVB on promoting older adult’s health should be further investigated using a larger-scale RCT, combined with follow-up measures in the community. Health practitioners may consider LVB as an adapted physical activity intervention to promote health outcomes in older adults in the future.

### Objectives

In response to the promising results of the LVB pilot study and the priority of allocating resources to the prevention of age-related fitness degradation in HKG, the present project aims to extend the aforementioned work, which is to investigate the effectiveness of a LVB intervention on physical and psychological health attributes among older adults in HKG. Specifically, the objectives of this study are:

1. compare the effects of a 16-week light volleyball (LVB) intervention program, with a TC program in terms of improving functional fitness among Chinese older adults aged 65 years or above;
2. compare the effects of a 16-week LVB intervention program, with a TC program in terms of improving psychological attributes (i.e., resilience, physical activity enjoyment) among Chinese older adults aged 65 years or above;
3. compare the effects of a 16-week LVB intervention program, with a TC program in terms of improving quality of life and balance among Chinese older adults aged 65 years or above; and.
4. evaluate the effectiveness of both interventions from the participants’ perspective in order to gain insight into the links between intervention elements and outcomes (e.g., identify mediators and moderators of program success).

## Methods

### Study design

This study utilizes both quantitative and qualitative methods with a larger sample (approximately 315 participants). We will adopt a RCT design to evaluate the effectiveness of the LVB intervention on health outcomes against an active comparison group (TC), and a control group. TC is chosen as our active control group because both LVB and TC are whole-body exercises, originating from China, and both are suitable for older adults and benefit older adults’ health [17, 18]. Next, compared to team-based LVB, TC is an individual PA that may impact the health and quality of life of older adults differently (e.g. perhaps psychologically). Evidence about the association between social support and PA suggests that older adults with greater social support (e.g. less social isolation) are likely to continue exercising [19]. Others [20, 21] demonstrated that increasing amounts of social support in community based group PA interventions were associated with increasingly beneficial effects (e.g., social functioning) relative to PA intervention and program adherence. Additionally, TC is a very popular activity among Chinese older adults [7]. When LVB is promoted or becomes popular in Hong Kong communities on a larger scale during the later phases of this project, TC is a good candidate for the PA utilized by the active control group in the current study. Perhaps a comparison between LVB and TC may provide some insight about PA promotion among HKG older adults in the future. We hypothesize that the LVB and TC interventions will produce significant improvements in selected physical and psychological health attributes compared to the control group. We expect the LVB intervention to be at least as effective as the TC intervention in enhancing health outcomes in older adults.

### Study intervention (objective #1–3)

In order to answer project objectives (a)-(d), the study will adopt a RCT. About 315 participants will be randomly assigned into LVB group, TC group and control group in 1:1:1 ratio; this will be about 103 participants in each group. Data will be collected at baseline (pre-test), after a 16-week intervention (post-test), and 6 months and 12 months after the intervention is completed. The intervention will be based on the previous program specifically designed for older adults in LVB [17] and in TC [18]. Each program consists of 32 training sessions, with 2 × 90-min sessions per week. The duration of the proposed intervention is consistent with the recommendation of U.S. Department of Health and Human Services (HHS) for PA recommendations [22]. The LVB Group’s program will be delivered by registered coaches from the Light Volleyball Association of HKG (LVBAHK). Content for this program is designed by the project co-ordinator (PC) and LVBAHK, which was found to be effective for improving health of older adults [17]. The TC Group will utilize a simplified 24-form Yang Style TC that is designed for easy learning and mastery. Participants will be led by a qualified TC instructor, and they will replicate instructor’s motions, postures, and movement speed. Any incorrect performance will be rectified by the instructor in the class. During the intervention, the control group participants will be instructed to maintain their normal daily activity and join regular non-exercise social gatherings in order to balance the psychosocial effect (i.e., to maintain the internal validity of the study) of regular gatherings for the LVB and TC groups.

### Participants

Inclusion criteria for participants will be (a) aged 65 years and above; (b) living in the community independently; (c) absence of diagnosed cognitive impairment; and will have (d) no participation in a structured PA program for two years preceding the study; and (e) passing score on the Timed-up-and-go test (TUG) and Abbreviated Mental Test (AMT). The TUG [23] and the AMT [24] are to assess participant’s physical competence and cognitive functioning, respectively. Participants i) whose time exceeds 20 s on the TUG or ii) whose AMT score is less than 6 or iii) who exhibit steady hypertension (160/90 mmHg or above), arthritis, and/or neurological disorders, will be excluded from the study.

### Sample size calculation

We computed a projected sample size using a statistical power analysis by G power [25]. The analysis was based on effect size (Cohen’s d = 1–1.88) from a previous study showing significant improvement in functional fitness (Cohen’s d = 1.88) and physical activity enjoyment (Cohen’s d = 1) following a LVB intervention, a control, and a comparison group [17]. With a more conservative effect size (Cohen’s d = .5), a total sample of 237 older adults is required in this three-group design study in order to achieve a power of 80% at a significance level of 5%. Finally, we aim to recruit 315 participants (45 participants per center, 7 elderly centers are needed, 105 participants in each group) in total, with a 25% expected dropout rate [26].

### Recruitment and procedures

**Fig. 1:**
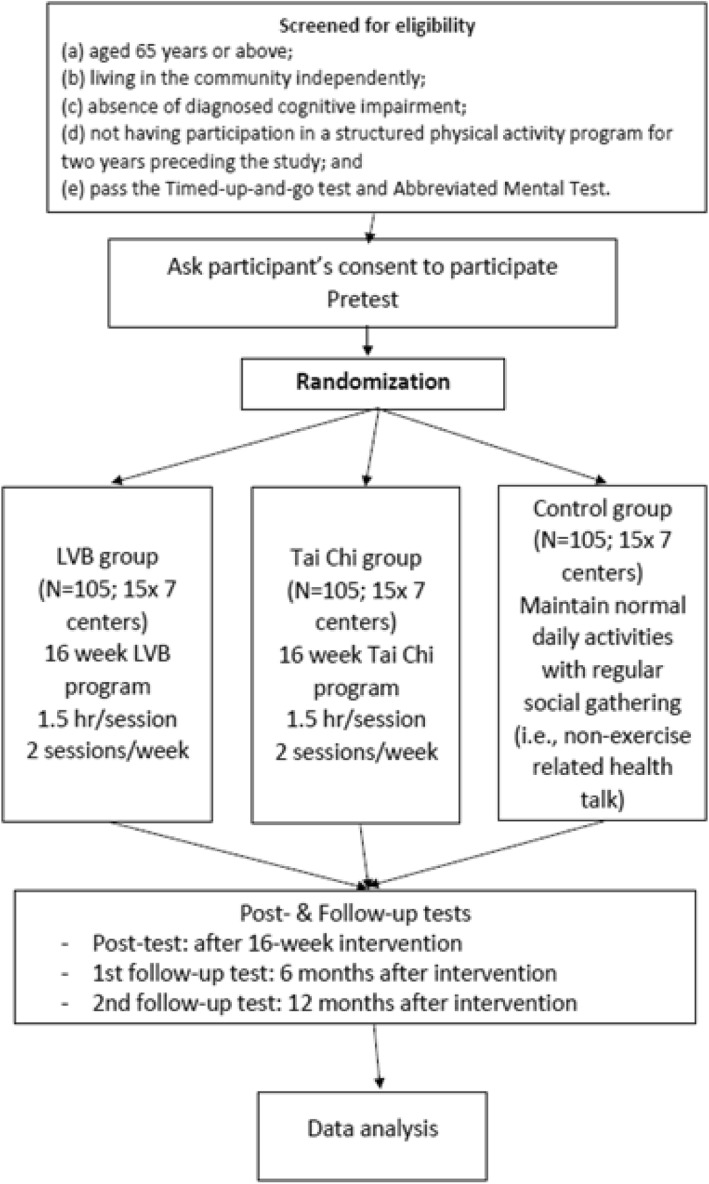
Design of the study with CONSORT guidelines of RCT

Participants will be recruited via a presentation offered by the research team and via advertisements in the local neighborhood elderly centers (NECs). An information session (e.g., aims and procedures of the intervention) will be delivered to groups of potential participants. Participants will be advised of the confidentiality of personal data and informed that they can voluntarily withdraw from the project at any time without prejudice. Upon agreement from participants, data collection will be conducted in person by a research assistant and trained student helpers after obtaining informed consent. At the first testing session, participants will do the screening test (i.e., TUG, AMT). Prior to the physical measures (pre-test), participants will complete the questionnaires, socio-demographic questions, height, weight and percentage of body fat using a Tanita machine (model: TBF-410GS). Participants will then take the functional fitness tests in the standardized order used in Leung et al. [17]. The intervention will begin in the second week and continue as described. Participants will complete the post-test (same as the pre-test) within 7 days of following the 16 week (4 months) intervention. As suggested by Fjeldsoe et al. [27], two follow-up tests will be done 6 months and 12 months after completion of the intervention. All participants will receive a HK$100 (US$12) supermarket cash voucher as incentive for their participation. *See Fig. 1 for the design of the study using the CONSORT guidelines for RCT.

### Measures

#### Physical attributes

##### Functional fitness

The Senior Fitness Test Manual [28] will guide the measurement of physical attributes of the participants. The tests consist of seven items: chair stand test (lower body strength), arm curl test (upper body strength), chair sit and reach test (lower body flexibility), back scratch (upper body flexibility), 8-ft up-and-go test (agility and balance), 2-min step test (aerobic endurance), and body mass index (BMI; kg/m2). These tests were found to be reliable (ICC: .80–.98 for participants in trials) and valid through content, construct and criterion-related analyses [28].

##### Balance test

The Balance System SD (Biodex) will be used to measure balance ability of participants in our intervention study. This system has previously been used to assess balance in a group of Hong Kong Chinese older adults [29]. In the test, participants will be asked to maintain the vertical projection with their centre of gravity in the centre of the platform by observing a vertical screen located 30 cm in front of their face. Each assessment will take 20 s, with 10-s rest periods in between. The average of the results from three trials will be obtained. Reliability (test-retest reliability = .69–.80) and validity of the balance test was supported in the studies of Parraca et al. [30] and Finn et al. [31].

#### Psychological attributes

##### Resilience

The 25-item Resilience Scale (RS) [32] will be used to measure resilience (i.e., the outcomes of successful adaptation despite challenging or threatening circumstances of participants). This scale is reliable (test-retest reliability = .8; Cronbach’s α = .95) for Chinese elderly populations [33]. The exploratory principal component analysis also found that RS was valid in a four-factor structure.

##### Quality of life

Quality of life will be measured using the Chinese version of the Medical Outcomes Survey 36-Item Short Form Health Survey (SF-36) [34]. The reliability and validity of the SF-36 questionnaire have been confirmed for Chinese individuals [34]. This survey contains eight domains: physical functioning (PF), physical role, bodily pain, general health (GH), vitality, social functioning (SF), emotional role, and mental health. In the present study, only PF, SF and GH will be included in order to lower the participants’ burden to complete the questionnaires. Examples of the items include asking participants if they encountered any restrictions or limitations while performing moderate PA, such as moving a table, using a vacuum cleaner, bowling, playing golf, and walking. The score ranges from 0 to 100 and a higher score indicates better physical functioning.

##### PA enjoyment

The short version (8 items) of the PA Enjoyment Scale (PACES) [35], translated to Chinese, will be used to measure participants’ PA enjoyment throughout the study. The Scale was originally developed by Kendzierski and DeCarlo [36]. This short version the PACES questionnaire is a reliable and valid instrument for assessing PA enjoyment in Chinese older adults [35]. In specific, the test-retest reliability and internal consistency was moderate (intraclass correlation coefficient = 0.614) and high (Cronbach’s alpha = .91–.92), respectively. Its convergent validity was supported that PACES was moderately correlated with quality of life in older adults.

### Data analysis

Data will be analysed using SPSS 24.0 with a significance level of .05. To answer study objectives (a)-(c), generalized estimating equations (GEE) models will be used to analyse mean changes in outcomes over time among the three groups with adjustment for baseline characteristics showing statistically significant differences among the groups. GEE models was used in similar study such as Kekäläinen et al. [37] and it take into account the Intra-class correlation coefficient of responses within an elderly centre and correlations between repeated measurements in the same individual. In addition, the GEE models do not require complete data and can be fit even when individuals do not have observations at every time point. Descriptive statistics of the socio-demographic variables (e.g., frequency, M and SD) will also be computed.

### Qualitative study (objective #4)

#### Participants

In order to identify the effectiveness of the intervention from the participants’ perspective and make recommendations for maximizing retention and developing future interventions and maximizing retention, thirty participants from LVB group will be recruited purposively, stratified by gender and intervention completion (vs non-completion) of intervention. A participant who has attended at least 80% of the sessions [38] will be considered an intervention completer.

#### Procedures

LVB participants in the RCT will be invited to join a qualitative arm of the study. Semi-structured group interviews (groups of 4) will be used to collect information on participants’ (a) experiences and thoughts about the program, (b) comments about the design of the intervention, and (c) changes in their PA engagement, and physical and psychological changes in relation to the intervention program. Interview questions will be based on the literature related to PA interventions from a Social Ecological perspective [39]. These questions will be pilot-tested on 10 older adults and investigators involved in the study.

### Data analysis

All interviews will be transcribed verbatim and verified. To facilitate data reduction, qualitative data analysis software QSR-NVivo will be used for thematic coding. During coding, data will be organized into conceptual categories/themes based on the Social Ecological Model (SEM) (e.g., individual, interpersonal, organizational, community, and policy levels of impact) [40]. Two independent coders will read, re-read, and code a portion of the interview transcripts independently to ensure that coding is done in accordance with the research questions, and to triangulate data analysis. Lastly, coded transcripts will be compared and discussed among the coders and investigators again to further facilitate the development of themes related to the SEM.

## Discussion

This RCT and qualitative study aims to investigate the effectiveness of a LVB intervention on physical and psychological health attributes among older adults in HKG. We hypothesize that the LVB and TC interventions will produce significant improvements on the selected physical and psychological health attributes compared to the control group. We expect the LVB intervention to be at least as effective as the TC intervention in enhancing health outcomes in older adults. The expected and direct beneficiaries are expected to be:

***Older adults in HKG*** will benefit from enhancing their physical and psychological health after participating in a 16-week LVB intervention. Also, they will benefit from improved service delivered to the older adults through non-government organizations that promote LVB. Older adults in control group, even not involving in PA will also increase the understanding about their own fitness level by doing fitness tests (both short and long term);

***Non-government organizations (NGOs)*** will benefit because they will have access to information and resources related to a newly developed PA, LVB. These NGOs will also benefit by enhancing their members’ health outcomes as a result of participation in the project. The results of the qualitative study will also provide evidence-based information (e.g., workforce, service delivery) to practitioners to achieve a more effective organization of LVB and other PA programs in the future. Finally, this collaborative project will increase the NGOs’ awareness of promoting healthy ageing (e.g., LVB or other programs) through more effective and active community engagement (both short and long term).

***Government departments*** such as the Food and Health Bureau, and Leisure and Cultural Services Department in Hong Kong, will benefit from the data and evidence to support their policymaking in relation to PA promotion for older adults. Policymakers will benefit from working together to provide quality and sustainable health services to the public, and in return, the healthcare expenditures will be reduced in HKG government. Policymakers will also have a better understanding of the effectiveness and efficiency of a PA promotion program such as which elements would work better for older adults. On a macro scale, this project may inform HKG government about the ways of promoting healthy ageing using promotion of LVB as an example of moving from mass participation, to regular practice and training, and to sport competitions (e.g. in the Master Games) in the future (long-term impact).

***LVB and Volleyball associations in HKG*** will benefit from promoting LVB in HKG. Very likely LVB will be further promoted in other countries of the world (both short and long term).

## Data Availability

The datasets generated and analyzed during the current study are not publicly available due to ethical restrictions but are available from the corresponding author upon reasonable request.

## Abbreviations

AMT: Abbreviated mental test
BMI: Body mass index
GEE: Generalised estimating eqs.
GH: General health
HKG: Hong Kong
HKSAR: Hong Kong Special Administration Region
ICC: Intra-cluster correlation coefficient
LVB: Light volleyball
LVBAHK: Light Volleyball Association of Hong Kong
NECs: Neighbourhood elderly centers
NGOs: Non-government organizations
PA: Physical activity
PACES: Physical enjoyment scale
PC: Project-coordinator
PF: Physical functioning
RCT: Randomised controlled trial
RLA: Rouliqui; RS: resilience scale
SEM: Social ecological model
SF: Special functioning
TC: Tai chi
TUG: Timed-up-and-go
WHO: World Health Organisation
